# Altering The Primal Environment: Health Effects Associated With Assisted Reproductive Technologies

**DOI:** 10.1289/ehp.120-a390

**Published:** 2012-10-01

**Authors:** Julie Halpert

**Affiliations:** **Julie Halpert** is a Michigan-based freelancer writer. She has covered science, health, and the environment for numerous national publications over the past two decades, including *The New York Times*, *Newsweek*, *Yale Forum on Climate Change & the Media*, *Scientific American*, and *Technology Review*. She also teaches an environmental journalism class at the University of Michigan.


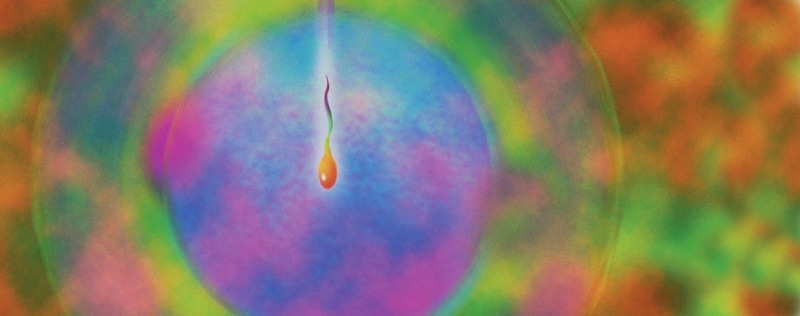
When Darine El-Chaar began her residency in obstetrics and gynecology at the University of Ottawa five years ago, she grew curious about the potential health repercussions of assisted reproductive technologies (ART), the catchall term for procedures used to help couples artificially conceive a child. ART involves surgically removing eggs from a woman’s ovaries, combining them with sperm in the laboratory, and returning them to the womb.^^1^^ Women undergoing ART take “fertility drugs” such as clomiphene citrate and gonadotropins to stimulate the production of many eggs rather than the single egg that would normally grow during their monthly menstrual cycle.^^2^^

El-Chaar wondered about the influence that ART procedures, as well as the underlying infertility itself, might have on the health of children conceived. She is one of many researchers working to answer the fundamental question of whether introducing fertility drugs and manipulating eggs and sperm in a laboratory setting—in essence, altering the primal environment—sets the stage for adverse health effects in children.

**Figure:**
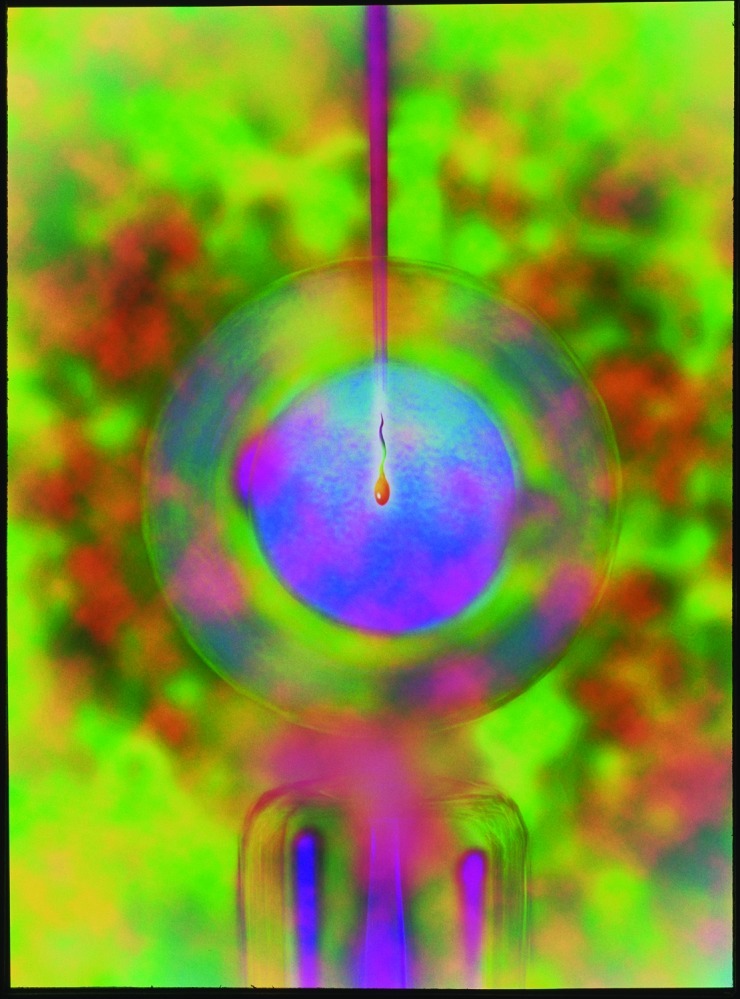
Artist’s rendering of intracytoplasmic sperm injection, in which a single sperm is inserted into an egg. © Mehau Kulyk/Photo Researchers, Inc.

Although some research indicates such a risk exists, there haven’t been enough large-scale studies to ascertain if the potential effects are severe enough over the long term to deter couples from seeking infertility treatments. Meanwhile, some findings are pointing the way toward possible refinements to improve health outcomes for ART babies.

## What Is ART?

After Robert G. Edwards and Patrick Steptoe developed the *in vitro* fertilization (IVF) procedure that produced the first “test tube baby” in 1978, it didn’t take long for ART usage to gain purchase in the medical world. In 2009 more than 60,000 babies were born through ART in the United States, up from 5,194 in 1990.^^3^^ And worldwide the total number of babies ever born through IVF through 2011 is estimated at 5 million.^^4^^ ART now represents a multibillion-dollar industry,^^5^^ with patients paying an average of $12,400 per treatment cycle.^^6^^ Simon Fishel, a professor and managing director of the Centres for Assisted Reproduction (CARE) Fertility Group in the United Kingdom, who worked with Edwards and Steptoe to produce that first successful IVF birth, says, “We knew its relative potential, probably just not the extent we have reached in the space of time it has taken. It still blows me away!”

**Figure:**
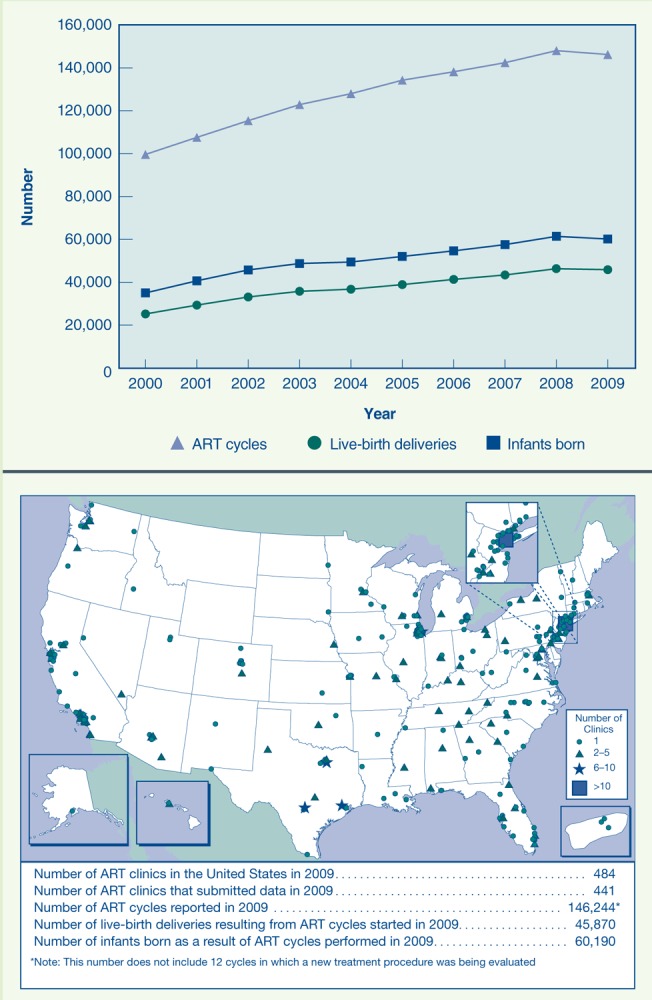
The number of ART births has increased steadily since 2000, with more than 60,000 ART-conceived infants born in the United States in 2009. (There are fewer live-birth deliveries than infants born because each set of multiples counts as one delivery.) CDC/ASRM/SART. 2009 Assisted Reproductive Technology Success Rates: National Summary and Fertility Clinic Reports. Atlanta, GA:Centers for Disease Control and Prevention (2011). Available: http://www.cdc.gov/ART/ART2009 [accessed 15 Sep 2012].

The IVF that Edwards and Steptoe pioneered has changed markedly since 1978 but remains the most popular form of ART, accounting for 99% of procedures.^^6^^ It is used to treat infertility caused by a broad range of factors, from blocked fallopian tubes, to endometriosis, to male-factor infertility.

Eggs retrieved during an outpatient surgical procedure are combined with sperm in a petri dish; then the resulting embryo is placed in the woman’s uterus in the hope it will implant. To increase the odds of an effective pregnancy, it’s not unusual to implant more than one embryo, which is why the procedure can lead to multiple births.^^1^^

Intracytoplasmic sperm injection (ICSI), first performed in Belgium in 1991, is a type of IVF developed to address problems with sperm quality such as reduced motility or very low sperm count. Today it is performed in roughly 60% of ART cycles in the United States.^^1^^ In this procedure, an individual sperm is injected through a microneedle directly into an egg. Multiple eggs are fertilized, and then one or more embryos are placed into the uterus, with the number depending on the woman’s age and the quality of the eggs.^^1^^

Advances in IVF’s effectiveness and its lower cost have pushed other technologies, such as gamete intrafallopian transfer (GIFT) and zygote intrafallopian transfer (ZIFT), out of favor. GIFT does not require that embryos be cultured; instead, a mixture of eggs and sperm is placed into the fallopian tubes, and fertilization occurs in the uterus. ZIFT differs from GIFT in that the egg is fertilized in the laboratory before being inserted into the fallopian tubes. Both can be used for women with cervical blockages, says David Frankfurter, medical director of IVF at The Medical Faculty Associates, George Washington University School of Medicine, and GIFT addresses religious objections to creating life outside the womb. But unlike IVF, both GIFT and ZIFT require laparoscopic surgery, introducing the potential for more complications.^^1^^

ART treatments can involve either fresh or frozen embryos. El-Chaar says fresh embryos are usually cultured until five days, then transferred to the womb, whereas frozen embryos can be stored for one to three years. Typically, fresh embryos are used for a couple’s first ART cycle, and the remaining embryos are frozen for use if needed. Some couples may freeze embryos for special circumstances, such as when one of the individuals is facing cancer treatment that may affect fertility.

## Health Questions

One of the chief health-related questions about ART is whether these procedures contribute to birth defects. A May 2012 study examined birth defect incidence in nearly 309,000 Australian children, 6,163 of whom were conceived through ART. The investigators found that birth defects occurred in 9.9% of babies conceived using ICSI, 7.0% of babies conceived using IVF that didn’t involve injection of the sperm into the egg, and 5.8% of babies conceived naturally.^^7^^ Overall, the researchers found a 26% increase in risk of birth defects among babies conceived using any form of ART (8% of births) relative to babies conceived naturally (6% of births), after adjusting for several factors that can influence birth outcomes.

The Australian study findings corroborated a 2005 review in which two-thirds of the studies assessed showed a 25% or greater increase in risk of birth defects in ART infants compared with naturally conceived infants.^^8^^ After pooling the results of all 25 studies reviewed, the relative increase in risk of birth defects was about 30%, whereas pooling the results of a subset of 7 studies considered appropriate for meta-analysis yielded a relative increase in risk of 40%. But lead author Michèle Hansen, a research officer at the Telethon Institute for Child Health Research in Perth, Western Australia, stresses that the “vast majority of children conceived following ART treatment are born healthy and do not have any birth defects.”

Other studies have looked at whether adverse outcomes are linked to the use of fresh versus frozen embryos. In 2011 investigators reported a 35% increased risk of low birth weight among singletons following fresh-embryo transfer, compared with frozen-embryo transfer, among nearly 57,000 IVF-conceived infants born in the United States between 2004 and 2006.^^9^^ They also found that fresh-embryo transfer was more likely to result in multiple births. A 2012 report described similar results and furthermore observed that the brand of culture medium used also appeared to influence how well fresh and frozen embryos fared.^^10^^

Hansen says there is evidence that use of fertility drugs alters endometrial receptivity, the period of time when embryos are able to implant in the wall of the uterus. She says this may partly explain why better outcomes are being reported for children born following frozen-embryo transfers (which do not involve the use of such drugs) compared with fresh-embryo transfers (which do). However, she points out these outcome data largely refer to embryos created with traditional slow-freezing methods, not the newer method of vitrification.

**Figure 2:**
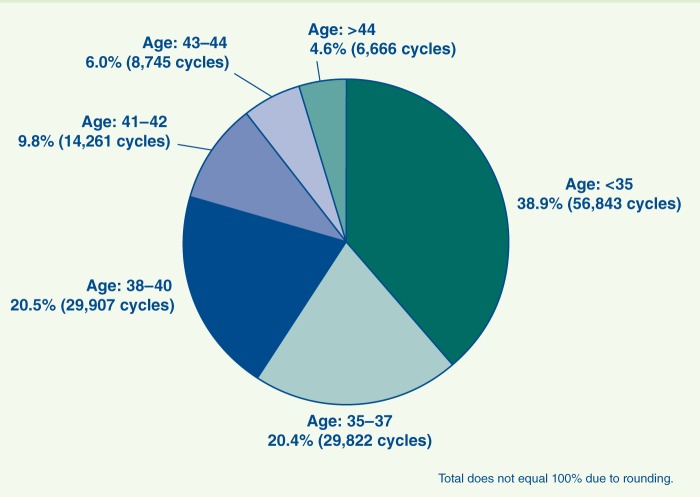
Most women who pursue ART are over age 35, which introduces its own set health risks to mother and child. CDC/ASRM/SART. 2009 Assisted Reproductive Technology Success Rates: National Summary and Fertility Clinic Reports. Atlanta, GA:Centers for Disease Control and Prevention (2011). Available: http://www.cdc.gov/ART/ART2009 [accessed 15 Sep 2012].

El-Chaar says doctors historically have often implanted more than one embryo at a time to improve the odds of success during the first try. This is important to many patients, most of whom bear the entire cost of treatments. But the multiple births that often result have been consistently linked with poorer health outcomes than singleton births—a phenomenon that is true of naturally conceived as well as ART births.

Infants conceived through ART are well known to have a higher risk of adverse outcomes such as low birth weight, preterm birth, and perinatal death.^^11^^^^,^^^^12^^^^,^^^^13^^ A few studies have shown increases in cerebral palsy following ART, especially in multiple births but also in singletons who are survivors of a vanishing twin (that is, one of the fetuses in a twin pregnancy dies *in utero*, and its tissues are absorbed by the other twin, the mother, or the placenta).^^14^^^^,^^^^15^^ Singletons who survive vanishing twins may also be more likely to have problems like those typically seen in multiples, including lower birth weight.^^16^^^^,^^^^17^^ “It appears that vanishing twin pregnancies resulting from fertility treatment—IVF or other treatment—do worse then singleton pregnancies, leading some to argue that vanishing twins may serve as a confounder in the IVF singleton data,” says Frankfurter. “This may account for some of the adverse outcomes seen with IVF singletons.”

Hansen says moving toward the use of single-embryo transfer could significantly reduce cerebral palsy rates in ART-conceived infants. Unpublished findings presented at the 2012 annual meeting of the European Society of Human Reproduction and Embryology suggest such a move has already improved health outcomes for ART-conceived babies in Australia.^^18^^

Another area of research involves certain childhood cancers. A 2012 report of 1,091 Greek and Swedish children with leukeumia or lymphoma showed a slightly elevated risk of leukemia following IVF. According to the result, 3% of Greek leukemia cases and 2.7% of Swedish leukemia cases were conceived through IVF compared with 1.8% and 1.6% of controls, respectively.^^19^^ However, no association was seen between IVF and lymphoma. In a study of 764 French children with acute leukemia, children were more than twice as likely to have been diagnosed with cancer if their mothers were treated with fertility drugs, but there was no such association for IVF.^^20^^ The authors suggest this may be due to the use of antiestrogenic drugs as the first-line therapy for the main causes of female infertility, whereas IVF involves treatment with other types of drugs. Lead author Jérémie Rudant says more research is needed to better quantify any relationships between specific types of fertility drugs and the role that underlying infertility could play in childhood leukemia.^^21^^

## Epigenetic Effects

Other questions concern whether sperm that need to be inserted directly into eggs are more likely to be deficient in some way, leading to adverse health effects later in life. Fishel says ICSI can allow severely compromised sperm to fertilize an egg, which may cause problems in the future offspring. “If there are gene issues, epigenetic factors, autistic-related factors, fertility gene defects, et cetera, associated with the severely compromised sperm condition, these may be carried across when *in vivo* fertilization could not occur,” he says.

Several studies have examined links between ART and epigenetic marks. ART has been associated with methylation of genes specific to an imprinting disorder^^22^^ known as Beckwith-Wiedemann syndrome, an overgrowth disorder accompanied by an increased risk of childhood cancer.^^23^^ And in one 2009 study, Carmen Sapienza, a professor at the Fels Institute for Cancer Research and Molecular Biology at Temple University, and colleagues found differences in methylation of a subset of genes, depending on whether a child was conceived *in vitro* or *in vivo*.^^24^^ Several of the genes whose expression differed were implicated in obesity and type 2 diabetes.

Sapienza and his colleague, Christos Coutifaris, chief of the Division of Reproductive Endocrinology and Infertility and a professor of obstetrics and gynecology at the University of Pennsylvania, as part of clinical trials sponsored by the U.S. National Institutes of Health, are now looking at how oxygen levels in culture medium affect methylation. The study, called PhOx IVF (The Effects of Physiologic Oxygen Tension on Clinical *in Vitro* Fertilization Outcomes), is testing whether embryos cultured at physiological oxygen levels of 5% (comparable to the uterine environment) have better outcomes than those cultured at the oxygen level of 20% that’s typical of a laboratory setting.^^25^^ The investigators will also assess whether oxygen tension affects genes related to growth. If the 5% oxygen level is confirmed to result in better outcomes, adopting it as the standard of care could help reduce risks associated with ART, Coutifaris says.

Other studies have found no increase in imprinting disorders with ART. A 2012 study examined how well methylation levels correlated with gene expression in 147 participants in the Epigenetic Birth Cohort at Brigham and Women’s Hospital in Boston. Although the investigators found no significant differences in transcriptional levels at imprinting control regions between those infants conceived through ART versus those conceived spontaneously, they did observe differences in methylation levels.^^26^^

The study also looked at babies conceived when women took fertility drugs but did not undergo subsequent ART treatments. No disruption in methylation was observed in the imprinting control regions. Because imprinting disorders associated with developmental effects are so rare, very large studies are needed to better understand the true prevalence of adverse outcomes with fertility treatments, including ovulation induction alone—an area that hasn’t been extensively investigated, says first author Rebecca C. Rancourt, a postdoctoral research fellow at the Harvard School of Public Health and Brigham and Women’s Hospital.

## Neurodevelopmental Outcomes

Researchers are also examining potential links between ART and autism spectrum disorders (ASDs). A recently published study found no association between ART and ASDs among singleton births, although it did find a statistically significant association for multiples.^^27^^ The study population included 370 children diagnosed with an ASD (including 21 multiple births) and 1,901 controls (including 54 multiple births). Study coauthor Lisa Croen, director of Kaiser Permanente’s Autism Research Program, says the study was limited because of its small sample size of multiples, and the investigators did not have information on specific types of infertility treatments.

In July 2012 researchers at the Harvard School of Public Health reported that women aged 35 and older who took fertility drugs were twice as likely to have a child with a mild ASD as women in the same age group who used no infertility treatment.^^28^^ A history of infertility in itself was not associated with an increased risk of having a child with an ASD. The study population included women participating in the Nurses’ Health Study II; among the 1,021 women in the 35-and-up age group, 164 had children who had been diagnosed with an ASD.

A 2011 study also suggested there may be modest increases in ASD risk for female children associated with fertility drugs, perhaps as part of artificial insemination.^^29^^ But this and the 2012 Harvard study each found a slightly different association, “so we really need more work to determine what is going on,” says Kristen Lyall, lead author of the Harvard study and now a postdoctoral research fellow at the University of California MIND Institute. She says understanding the direct impact of fertility drugs is complicated by the fact that ASDs are likely caused by a variety of factors that differ for different cases. She adds, “It all needs to be weighed against other potential risks of infertility treatments and the fact that these procedures do really help a lot of couples achieve healthy pregnancies.”

Investigators at the Eunice Kennedy Shriver National Institute of Child Health and Human Development (NICHD) are spearheading the first large population-based study in the United States to look at neurodevelopmental effects of ART.^^30^^ New York State has a detailed system that requires birth certificates to indicate if the child was conceived using infertility treatment. The state also closely tracks the method of artificial conception. Researchers with Upstate KIDS (The Upstate New York Infant Development Screening Program) are following 6,100 infants (including about 1,100 sets of twins) born to nearly 1,300 mothers who underwent infertility treatments and 3,700 mothers who conceived naturally. The researchers are screening the children for cognitive and neurodevelopmental outcomes at various intervals from birth to 3 years, including screens for ASDs at 18 and 24 months of age.

“Kids from infertility treatments tend to be born earlier and have a low birth weight, so we want to see if they experience delays that are a result of their birth circumstances or that are unique to infertility treatment,” says Mary Hediger, a project officer on the study and deputy director of the NICHD Division of Epidemiology Statistics and Prevention Research. Researchers are finishing the last round of screenings, and results are due in a year and a half. Hediger says several ongoing studies in both the United States and Europe, including hers, are looking at the longer-term effects of infertility treatment. She expects those will provide results within the next five years that will help to answer many of the outstanding questions in this area.

## Long-Term Health for ART Babies

Sapienza says that although babies born through ART generally appear perfectly normal, they may have moderately increased risks for a number of the undesirable outcomes that low birth weight can perpetuate. For example, he says if babies’ growth is restricted, it may mean that the genes involved in the body’s ability to metabolize sugar could be affected, leading to potential problems with hypertension, obesity, and diabetes. He says many of these potential effects might not manifest until adulthood. Therefore, some researchers are exploring questions such as what happens when these people turn 50 and whether ART adults will be more likely to have diabetes.

Coutifaris says overall, the outcomes for adverse health effects of ART are still incredibly small. “Even if there is some underlying process that makes some of these [children] susceptible to certain conditions, the vast majority appear to be normal,” he says. Still, he says practioners should disclose these risks so patients can make an informed choice. Coutifaris believes that consent forms should clearly outline all potential risks. The Society for Assisted Reproductive Technology provides sample consent forms for doctors as well as a variety of recommendations for ensuring informed consent.^^31^^

Pasquale Patrizio, director of the Yale University Fertility Center, says more needs to be done to provide better baseline population data for study in the United States. Ideally he would like to see a general health registry that reports history of infertility and infertility treatment in order to compare rates of cancer in treated and untreated infertile women. He expects that the movement toward electronic medical records will allow for an easier, more effective way to merge and collect data for future evaluations of adults conceived through ART. Fishel says such studies will need to distinguish whether these adults are a product of a single- or multiple-embryo transfer, irrespective of whether they were a product of a multiple pregnancy.

Frankfurter says electronic data should be recorded in a way that can be used by physicians as well as by researchers, so it gets pooled through a larger information system, similar to the practices in European countries. Currently in the United States “there isn’t an effective way of discerning who had what fertility treatment and why,” he says. He hopes that with better ascertainment of data from both the IVF and non-IVF communities, “we will get better conclusions.”

As far as reporting results goes, Hansen points out it is difficult to assess safety in a field where techniques are changing so rapidly and where there is often a substantial delay before health outcome data are reported. For example, she says, the 2012 Australian birth defect study^^7^^ considered ART births from 1986 to 2002, which leaves 10 years of more recent data that are not included and that may, in fact, have a different birth defect prevalence due to changes in laboratory practices and patient mix over that time period. “This is not to play down the importance of the study in any way,” she says, “but simply to illustrate that researchers in this area are constantly playing a catch-up game, where new techniques are introduced just as we are able to begin reporting on health outcomes of older techniques.”

Frankfurter says that, although he isn’t convinced that serious risks associated with ART have been demonstrated, it’s helpful to keep in mind that infertility is considered a disease^^32^^ and that there are very few diseases that don’t have a consequence associated with them. “[ART] is the treatment of an illness, and for every illness that’s treated, the interventions have an upside as well as a potential downside,” he says. “IVF really should be seen as no different.”
